# From obscurity to urgency: a comprehensive analysis of the rising threat of duck circovirus

**DOI:** 10.1186/s13567-024-01265-2

**Published:** 2024-01-26

**Authors:** Xinnuo Lei, Anping Wang, Shanyuan Zhu, Shuang Wu

**Affiliations:** Jiangsu Key Laboratory for High-Tech Research and Development of Veterinary Biopharmaceuticals, Engineering Technology Research Center for Modern Animal Science and Novel Veterinary Pharmaceutic Development, Jiangsu Agri-Animal Husbandry Vocational College, Taizhou, 225300 Jiangsu China

**Keywords:** Duck circovirus (DuCV), epidemiology, infection, diagnosis

## Abstract

Duck circovirus (DuCV) is a small, nonenveloped, single-stranded DNA virus with immunosuppressive effects on ducks that leads to slow growth and elevated mortality following mixed infections. Its infection manifests as feather loss, slow growth, swelling of respiratory tissue, and damage to immune organs in ducks. Although single infections with DuCV do not cause noticeable clinical symptoms, its ability to compromise the immune system and facilitate infections caused by other pathogens poses a serious threat to duck farming. Given the prevalence of this disease and the increasing infection rates in recent years, which have resulted in significant economic losses in duck farming and related sectors, research and control of DuCV infection have become especially important. The aim of this review is to provide a summary of the current understanding of DuCV, serving as a reference for subsequent research and effective control of the virus. We focus mainly on the genetics and molecular biology, epidemiology, clinical symptoms, and pathology of DuCV. Additionally, topics such as the isolation and culture of the virus, vaccines and antiviral therapies, diagnostics, and preventative measures are discussed.

**Supplementary Information:**

The online version contains supplementary material available at 10.1186/s13567-024-01265-2.

## Introduction

Circoviruses are small, nonenveloped viruses with single-stranded circular DNA genomes. According to the International Committee on Taxonomy of Viruses (ICTV) report released in 2022, the *Circoviridae* family comprises two genera: *Cyclovirus* and *Circovirus*. The *Circovirus* genus infects various mammals, with notable examples including Porcine circovirus (PCV) type 1–4 in pigs, Canine circovirus (CCV) in dogs, and various strains in bats. Among these, PCV2 is particularly significant due to its role in porcine circovirus-associated disease (PCVAD), which can cause symptoms ranging from organ damage to fatal outcomes in pigs [1]. In avian hosts, circoviruses such as psittacine Beak and feather disease virus (BFDV), Goose circovirus (GoCV), and Duck circovirus (DuCV) can cause stunted growth, abnormal feathering, weakened immune systems, and vulnerability to other diseases. DuCV, which is especially prevalent in ducks, leads to growth restriction and immunosuppression, often followed by severe secondary bacterial infections, posing a significant economic challenge to the poultry industry [2, 3].

However, since its initial report in a six-week-old duck flock in Germany in 2003, DuCV has not received adequate attention, and subsequent related research has remained scant. While several routine artificial infection experiments have been conducted over the past decade, providing some evidence of DuCV’s in vivo infection and multiorgan damage, limited research has been conducted on its infection and pathogenesis mechanisms due to the lack of a suitable in vitro culture system. There is a notable lack of medications and vaccines specific for DuCV infection. This situation has led to widespread indifference in the industry regarding DuCV risk, which has in turn affected efforts towards its prevention and control.

In recent years, there has been an escalating focus on the prevalence of DuCV infections in duck flocks, particularly in the coastal provinces of China. Epidemiological surveys indicate that the percentage of DuCV-positive birds among these flocks is increasing annually, warranting the attention of both agricultural enterprises and researchers. Therefore, it is important to perform detailed research on DuCV, aiming to understand its epidemiological characteristics and pathogenic mechanisms. Developing effective prevention and control strategies is not only scientifically valuable but also crucial for the advancement of duck farming.

This article conducts a systematic review of the existing research on DuCV, primarily focusing on three aspects, genetics and molecular biology, epidemiology, and clinical symptoms, along with pathological characteristics. The paper further discusses virus isolation and cultivation, diagnostic and detection methodologies, and the scope of vaccines, antiviral drugs, and preventive measures. By systematically summarizing and analysing existing research insights, this article strives to present a more holistic understanding of DuCV and lay a scientific foundation for impending research and prevention initiatives.

## Genetics and molecular biology

### Genotyping and genomic structure

DuCV is a nonenveloped, single-stranded, circular DNA virus that exhibits icosahedral symmetry and a diameter of approximately 15–16 nm, making it the smallest known duck virus. Its nucleic acid length ranges from 1755 to 1996 nt [4–6]. According to a comprehensive genetic evolution analysis of DuCV, Fu et al. proposed in 2011 that DuCV be divided into two major subtypes: DuCV-1 and DuCV-2. The nucleotide sequence variation across these two clades ranges from 13.2% to 17.4%, with genome lengths spanning from 1988 to 1996. These genotypes can be further divided into DuCV-1a, DuCV-1b, DuCV-2a, DuCV-2b, and DuCV-2c [7]. In 2013, Zhang et al. introduced DuCV-1c to the classification system and determined that DuCV-2a is a recombinant originating from the rep gene isolates DuCV-1a and DuCV-2b [8]. In 2016, a new recombinant strain, YN26/2013, was isolated by Sun et al. from a duck in Yunnan Province, China; the complete genome is 1987 nt in length. Recombination between DuCV-1 and DuCV-2 occurred in the 987–1111 nt region of the YN26/2013 genome [9]. In 2020, Ji et al. identified six new strains and classified them as the DuCV-1d subtype. Recombination analysis revealed that this new subtype emerged from recombination between the DuCV-1 rep and cap genes [4]. In 2022, a new type, DuCV-3, was discovered in laying ducks in Hunan Province, China. Its genome size is 1755 nt, and it is associated with reduced or halted egg production. The genomic homology between DuCV-3 and the genomes of DuCV-1 and DuCV-2 range from 62.3% to 63.7%. Research on this newly identified subtype is limited [5].

**Figure 1:**
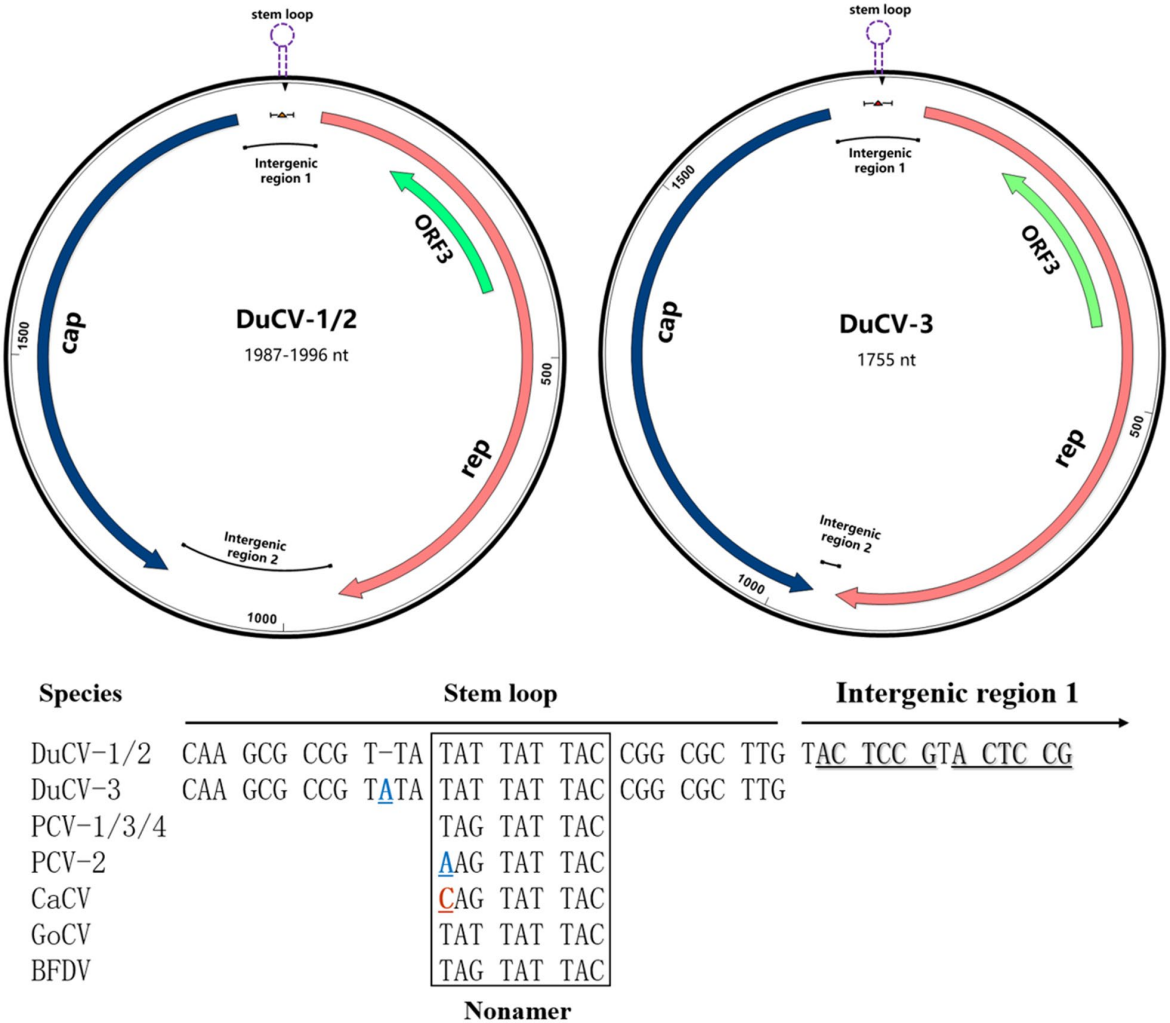
**Genome structure of DuCV and the nucleotide sequence of the stem‒loop region.** Significant differences exist in the total genome length and length of intergenic region 2 between DuCV-1/2 and DuCV-3. The nonamer in the stem‒loop shows some degree of conservation across different viruses.

The DuCV genome contains three major open reading frames (ORFs), namely, ORF1, ORF2, and ORF3, as well as two intergenic noncoding regions (Table 1 and Figure 1). The difference in total genome length between DuCV-1/2 and DuCV-3 is attributed mainly to the difference in the length of intergenic region 2. Intergenic region 2 in DuCV-3 is 197–206 nt shorter than that in DuCV-1/2, but how this difference impacts viral replication is unclear. Additionally, all intergenic region 1 sequences contain a stem‒loop, which includes a nonamer (Figure 1). This stem‒loop structure is conserved in all circoviruses and is used to initiate rolling-circle replication. However, the first three nucleotides of the nonamer may vary among different viruses (Figure 1). Adjacent to the stem‒loop on the right side, two hexamers, 5ʹ—ACTCCG, are found in intergenic region 1 as binding sites for rep gene products [6]. Interestingly, the region spanning the 3' ends of the rep and cap genes contains four repeats of a 44-base pair sequence, which is unique to DuCV [6].

**Table 1:** **Characteristics of different DuCV genotypes**.

Genotype	DuCV-1				DuCV-2			DuCV-3
Subtype	1a	1b	1c	1d	2a	2b	2c	N/A
Genome length	1995 nt	1996 nt	1995 nt	1987 nt	1988 nt	1988 nt	1988 nt	1755 nt
Stem loop	1977–1995 and 1–11	1978–1996 and 1–11	1977–1995 and 1–11	1969–1987 and 1–11	1970–1988 and 1–11	1970–1988 and 1–11	1970–1988 and 1–11	1736–1755 and 1–11
Nonanucleotide	1989–1995 and 1–2	1990–1996 and 1–2	1989–1995 and 1–2	1981–1987 and 1–2	1982–1988 and 1–2	1982–1988 and 1–2	1982–1988 and 1–2	1749–1755 and 1–2
ORF1 (*rep*)	49–927 (879 nt)	49–927 (879 nt)	49–927 (879 nt)	49–927 (879 nt)	48–926 (879 nt)	48–926 (879 nt)	48–926 (879 nt)	50–931 (882 nt)
ORF2 (*cap*)	1159–1932	1160–1933	1159–1932	1151–1924	1151–1924	1151–1924	1152–1925	958–1695
ORF3	164–400 (237 nt)	164–400 (237 nt)	164–400 (237 nt)	164–400 (237 nt)	163–399 (237 nt)	103–399 (297 nt)	103–399 (297 nt)	165–404 (240 nt)
Intergenic region 1	1933–1995 and 1–48	1934–1996 and 1–48	1933–1995 and 1–48	1925–1987 and 1–48	1925–1988 and 1–47	1925–1988 and 1–47	1926–1988 and 1–47	1695–1755 and 1–49
Intergenic region 2	928–1158 (231 nt)	928–1159 (232 nt)	928–1158 (231 nt)	928–1150 (223 nt)	927–1150 (224 nt)	927–1150 (224 nt)	927–1151 (225 nt)	932–957 (26 nt)
Refs. seq^a^.	MF627690	HM162351	GU014543	KR491946	EU344805	KP229377	ON227555	OP432310

^a^Reference sequences are based on Yuan et al. [39]. All gene characteristics are obtained from the analysis of reference sequences; however, information on other strains within the same subtype may vary. Data with significant differences are underlined.

### Structure and function of the encoded proteins

**Figure 2:**
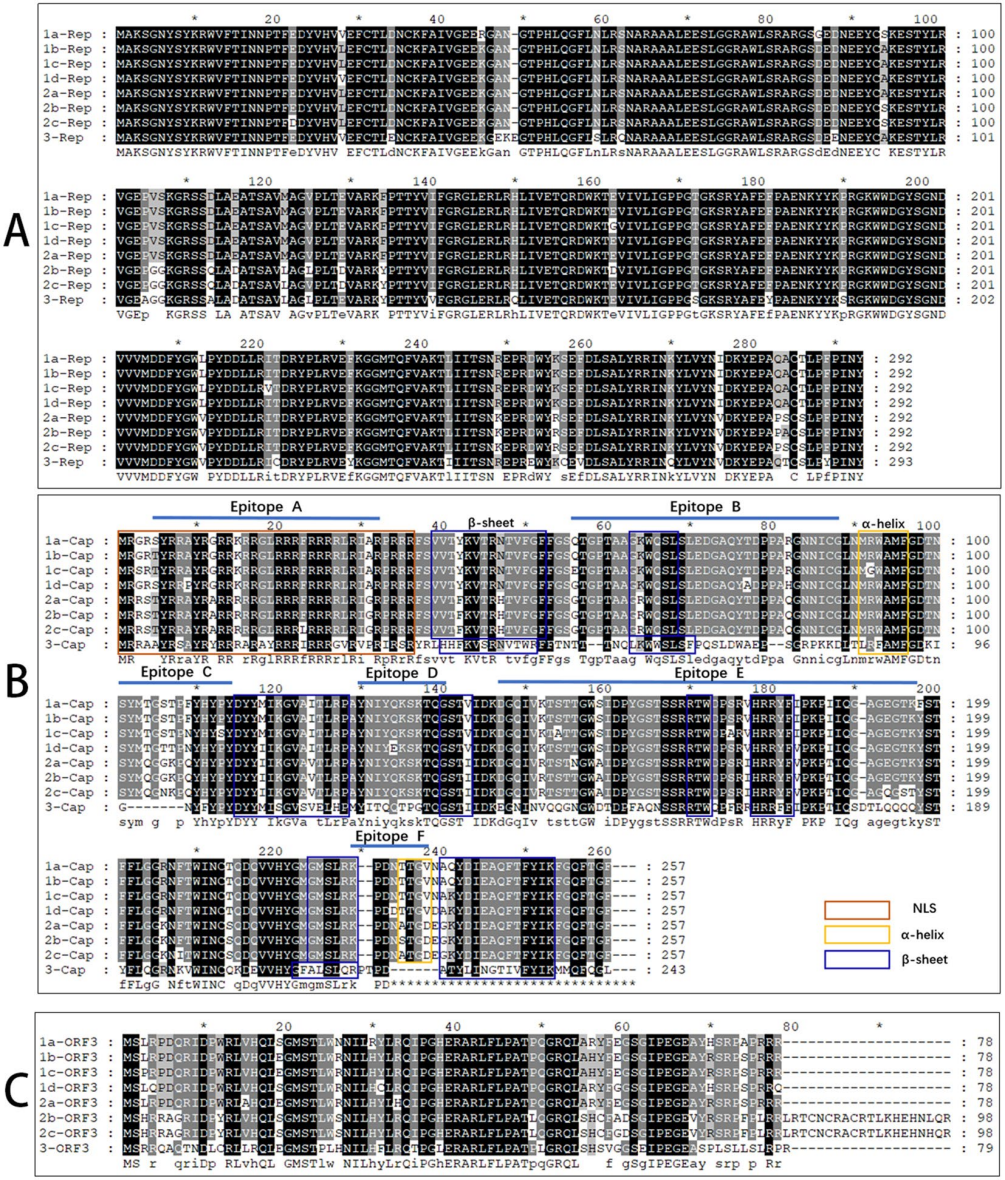
**Amino acid sequence alignment of proteins encoded by different genotypes of DuCV.** **A** Rep. **B** Cap, with the predicted nuclear localization signal and secondary structure annotated in different coloured boxes; areas of high antigenicity in 6 B-cell linear epitopes are also indicated (predicted by BepiPred-2.0). **C** ORF3. A black background indicates completely conserved sequences, and a grey background indicates highly conserved sequences.

The Rep protein is encoded by ORF1 and is essential for DuCV replication. The amino acid sequence of the Rep protein is highly conserved among various genotypes (Figure 2A). In DuCV-3, a mutation from “AN” to “EKE” at positions 48–49 leads to a change in the overall protein length from 292 to 293 residues. There have been no reported studies on the impact of this mutation on the function of the Rep protein. Along with other mutations in Rep, this results in a sequence identity ranging from 87.4 to 90.4% when compared to that of DuCV-1/2 [5]. The N-terminus of the Rep protein, specifically amino acid residues 10–37, contains a nuclear localization signal (NLS); deletion of these residues blocks nuclear translocation of the Rep protein [10]. However, further research is needed to elucidate the function and underlying mechanisms of the Rep protein in viral replication, which can also take advantage of cues from other viruses in the *Circovirus* genus, such as PCV. In PCV-1, transcription begins near nucleotide 767, and an intron (1176–1558) is spliced out, creating a shorter Rep’ protein that differs from the Rep protein due to a frameshift; importantly, PCV1 requires both Rep and Rep’ for replication initiation [11]. These viruses exhibit dual “nicking/joining” activities by cleaving the viral DNA strand between nucleotides 7 and 8 in the conserved nonanucleotide sequence at the peak of the stem‒loop structure. Additionally, they join viral single-stranded DNA fragments, contributing to the termination of viral DNA replication [12]. Rep’ forms a dimer through the interaction of its C-terminal domain, creating a positively charged groove, which is potentially crucial for the binding of double-stranded DNA in the virus [13]. However, whether the DuCV genome also expresses Rep’ and participates in viral replication has yet to be verified.

**Figure 3:**
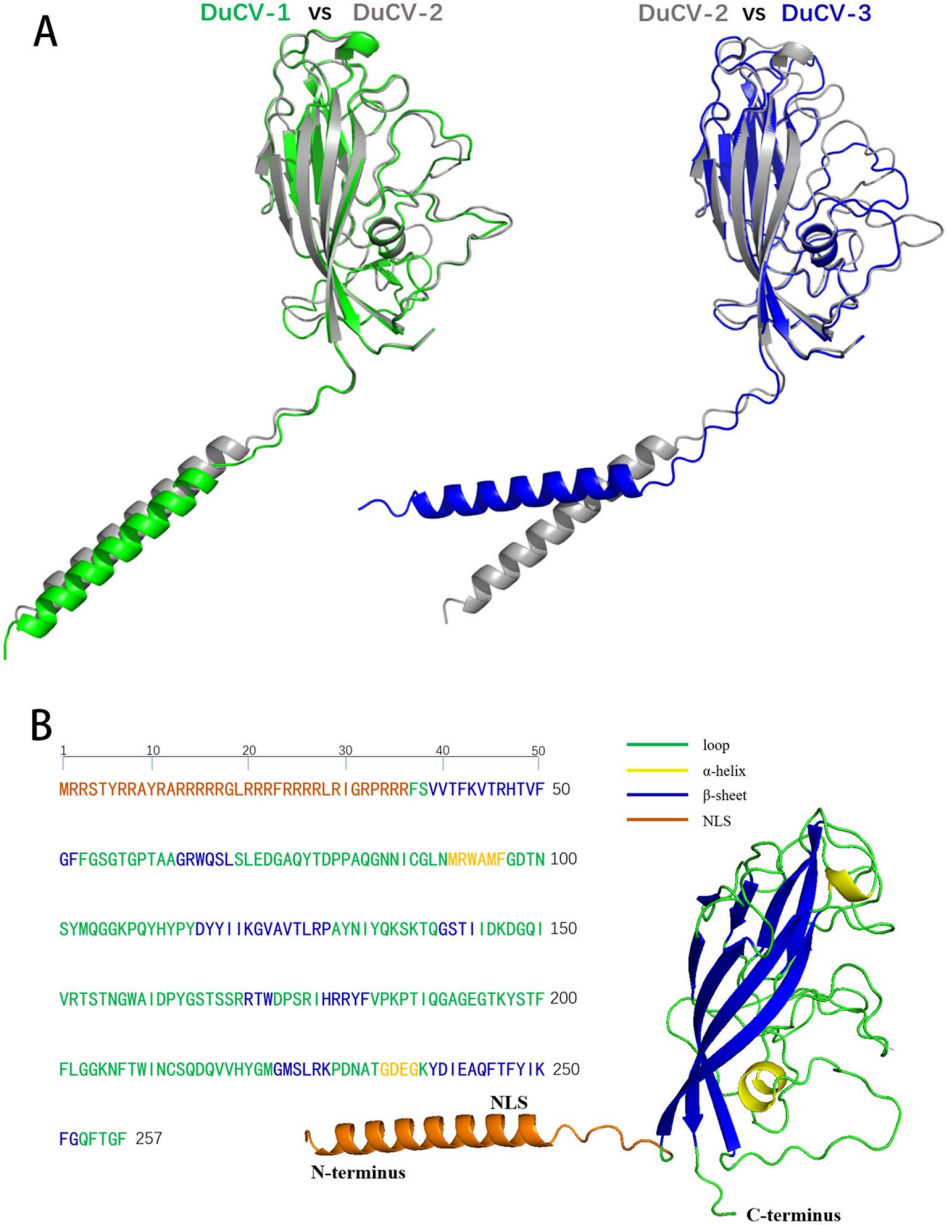
**Predicted structures of DuCV Cap across three genotypes**. **A** DuCV-1 Cap (green, reference sequence: HM162351), DuCV-2 Cap (grey, reference sequence: ON227555), and DuCV-3 Cap (blue, reference sequence: OP432310). **B** Analysis of the primary, secondary, and tertiary structures of DuCV-2 Cap. These structures were predicted using AlphaFold2.

The Cap protein, encoded by ORF2, is the only structural protein that forms the capsid of DuCV and has strong immunogenic properties. A complete DuCV viral particle exhibits icosahedral symmetry and consists of 60 Cap protein subunits. The amino acid sequence of the Cap protein is relatively conserved between DuCV-1 and DuCV-2, while that of DuCV-3 significantly diverges (Figure 2B). BLAST analysis revealed that the two strains have the highest identity (approximately 45%) compared to DuCV-1/2 or GoCV. However, the underlying cause of such substantial variation in the DuCV-3 Cap sequence is unclear. The structural differences among the Cap of DuCV-1, DuCV-3, and DuCV-2 are not significant (Figure 3A).

Due to the absence of crystal structure data for the DuCV Cap protein, its structure was predicted using AlphaFold2. Taking DuCV-2 Cap as an example, its amino terminal residue 1–36 serves as NLS. The main body structure likely contains two alpha helices and eight beta sheets, as well as numerous loops (Figures 2, 3B). These structural features of DuCV Cap resemble those of PCV Cap [14]. The abundant loops may hinder protein folding, which may be a major reason why Cap protein expression predominantly occurs in inclusion bodies in both prokaryotic and eukaryotic systems. Based on the structure of PCV Cap, it is hypothesized that the NLS at the amino terminus of the DuCV Cap is oriented towards the interior of the viral particle and that the abundant loop regions are located on the surface of the viral particle, where they can form antigenic determinants. To date, no information on the antigenic epitopes of DuCV Cap has been reported. The predicted B-cell linear epitopes (A–F) are located mainly in the loop regions (Figure 2B), and these predicted epitopes require experimental validation.

The DuCV ORF3 gene encodes a viral protein with apoptotic activity; this trait is common among ORF3-encoded proteins in the *Circoviridae* family, albeit with subtle variations in apoptotic activity depending on the cell type [15]. Initial verification of apoptotic activity was conducted in Sf9 cells infected with a recombinant baculovirus carrying DuCV ORF3. The percentage of apoptotic cells was significantly greater at 24, 48, and 72 h post-infection than among Sf9 cells infected with wild-type baculovirus [16].

Additional studies have shown that the DuCV-2 ORF3-encoded protein can induce chromatin condensation and fragmentation in duck embryonic fibroblasts (DEFs). This apoptotic effect is mediated by upregulation of cleaved caspase-3 and caspase-9, thus activating the mitochondrial apoptotic pathway in DEFs [17]. Compared to those of DuCV-1, the ORF3 proteins encoded by DuCV-2b and DuCV-2c are 20 amino acids longer at the C-terminus (Figure 2C). The extended sequence shows the highest identity (64%) with that of GoCV. Furthermore, the C-terminus contains a NLS peptide (RRLRTCNCRACRTLK), which is crucial for nuclear localization of the protein [18]. Deletion of these 20 C-terminal amino acids reduces the apoptotic activity of the protein [17]. Currently, it is unclear whether the ORF3-encoded proteins DuCV-1 and DuCV-3 also possess apoptotic activity and nuclear localization characteristics. Further studies are needed to determine the role of this protein in DuCV-1 and DuCV-3.

## Epidemiology

### Susceptible species, seasonal trends and transmission routes

The primary source of DuCV infection is domestic duck infection. Almost all breeds of ducks, including domestic ducks (Mulard, Aylesbury, Mule, Peking, Muscovy and Cherry Valley ducks) and wild ducks (Falcated, Mallard ducks, Velvet scoter and Green-winged teal), are susceptible to DuCV (Additional file 1). Moreover, there is no significant difference among DuCV strains infecting ducks from different species. Although DuCV infection can occur annually, infection rates tend to increase in summer and fall. Conversely, the detection rate of DuCV in dead ducks increases in winter and spring, likely due to reduced resistance in ducks caused by colder weather, leading to higher morbidity and mortality rates.

DuCV primarily propagates through horizontal transmission. While definitive studies are lacking, it is widely believed that DuCV may be transmitted through the cloacal-faecal-oral route. Furthermore, considering the detrimental effect of DuCV on feathers, the skin or feather base may also be plausible transmission routes. Notably, vertical transmission through eggs is also possible [19]. Ducks exhibit varying susceptibility to DuCV at different ages, with a pronounced incidence in duck flocks aged 3–5 weeks, for which morbidity rates range between 20 and 70% [20–23]. This heightened susceptibility might be linked to the decrease in maternal antibodies; ducks younger than 3 weeks have a decreased infection rate. However, as maternal antibodies decline and immune systems remain immature, the infection rate increases significantly in duck flocks aged 3–5 weeks and subsequently decreases as the immune system matures. In addition, environmental stressors such as relocation or increased flock density experienced by ducks aged 3–5 weeks may exacerbate their exposure risk to DuCV.

### Ancestral tracing of DuCV

**Figure 4:**
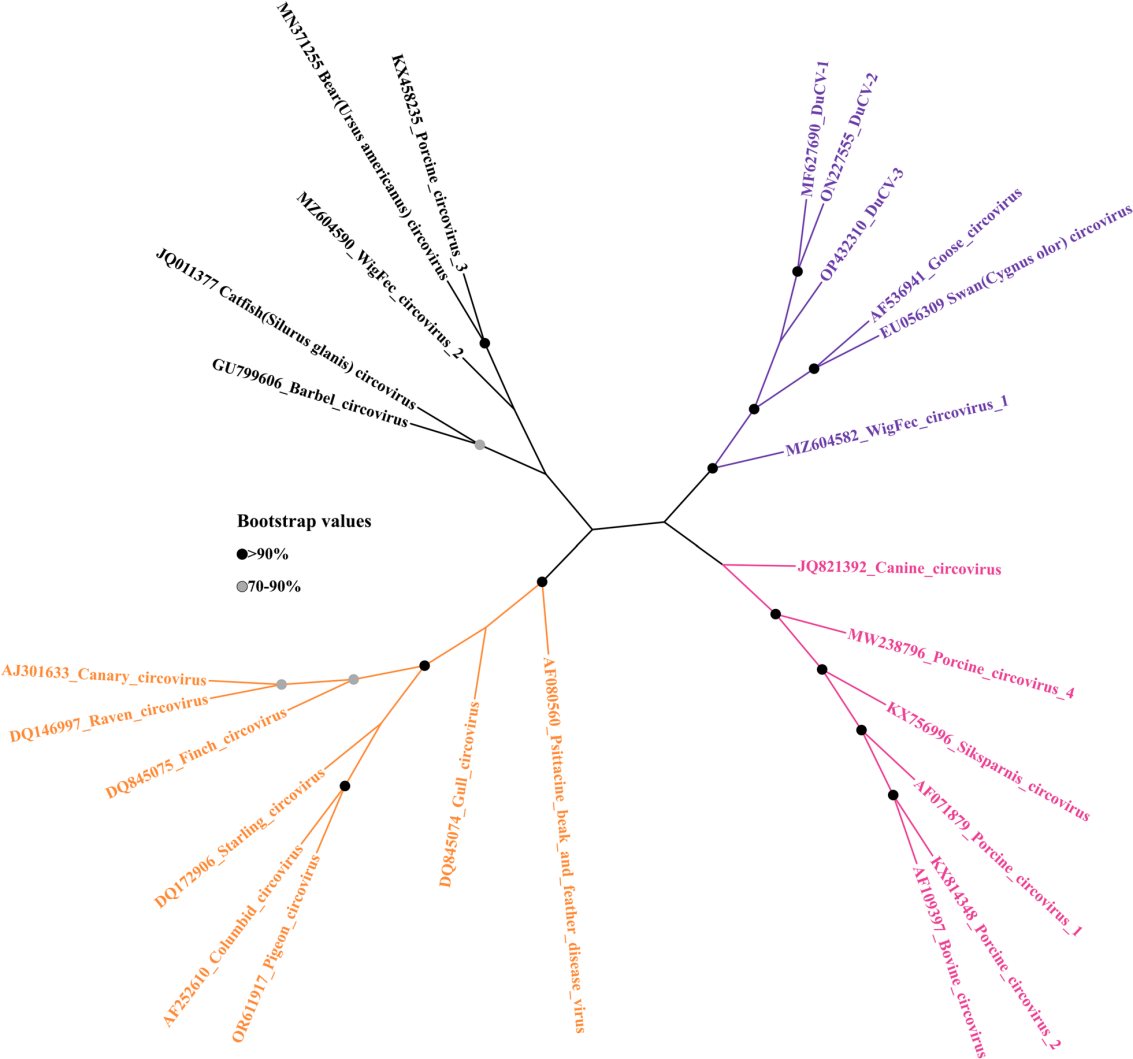
**Phylogenetic analysis of diverse circoviruses.** A phylogenetic tree was constructed through the application of the neighbour joining method within MEGA-11, employing complete genomic sequences of DuCV. A comprehensive bootstrap analysis with 1000 replicates was conducted to assess the robustness of the tree topology. The nodes are depicted as black circles when bootstrap values surpass 90% and as grey circles when bootstrap values range from 70 to 90%. Notably, these sequences can be effectively categorized into four distinct clusters, with branches in separate clusters delineated by distinctive colour codes to facilitate clear differentiation.

A phylogenetic tree constructed from the complete genomic sequence of the virus revealed the phylogenetic relationship between DuCV and other members of the *Circoviridae* family (Figure 4). These viruses are evidently classified into four distinct major clusters. DuCV, GoCV, Swan circovirus, and the recently discovered WigFec circovirus 1 identified in American wigeon all cluster together, indicating their close genetic relationship [24]. Further comparative analysis of the genomic sequences and major coding proteins of these circoviruses revealed sequence identities of 67.0%, 66.3%, and 55.6% for GoCV, Swan circovirus, and WigFec circovirus 1, respectively, with DuCV-1a. The highly conserved Rep protein amino acid sequences exhibits greater similarity, with percentages of 83.2%, 78.4%, and 63.9%, respectively. Conversely, the amino acid sequence of the Cap protein, which has a higher mutation rate, shares 47.1%, 55.5%, and 27.3% identity (Additional file 2). Notably, all four of these viruses have waterfowl hosts, implying possible common ancestry and the potential occurrence of genetic recombination events during their evolutionary history.

### Prevalence of DuCV in China

**Figure 5:**
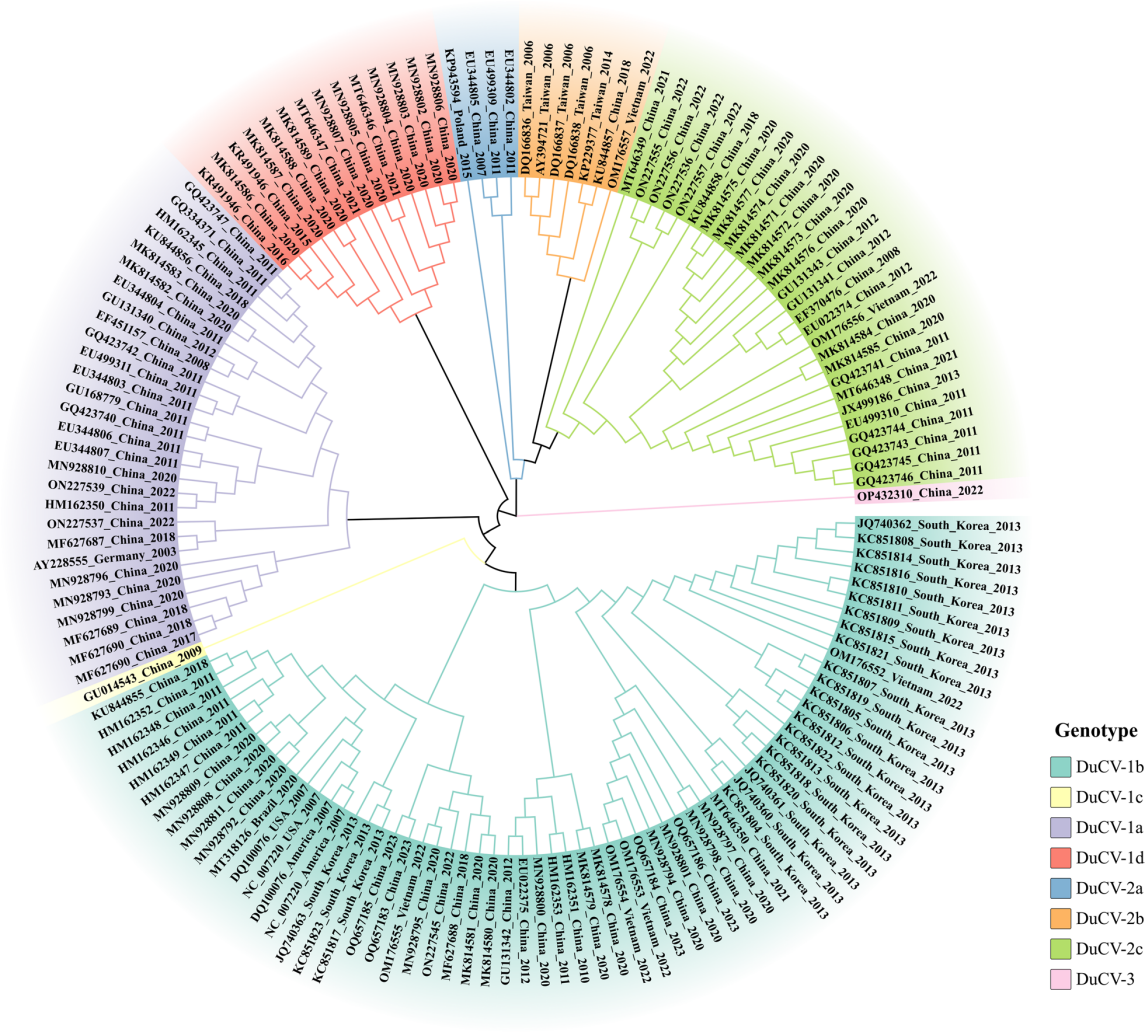
**Phylogenetic tree representing all genotypes of DuCV.** The phylogenetic tree was constructed using the neighbour joining method within MEGA-11, employing complete genomic sequences of DuCV. A bootstrap analysis with 1000 replicates was performed. Different genotypes are distinguished by distinct colours, as explained in the legend. All sequences were collected from the reports listed in Additional file 1.

DuCV was initially identified in two female 6-week-old Mulard duck flocks in Germany in 2003, with subsequent reports surfacing from Hungary, Taiwan, the United States, mainland China, and South Korea, etc., highlighting the global dispersion of DuCV [6, 20, 25–33]. According to the results of epidemiological investigations (Additional file 1) and phylogenetic tree analysis (Figure 5), the prevalence of DuCV genotypes shows a certain degree of regional variation. Different provinces in China exhibit distinct DuCV genotype prevalence patterns, while other regions are associated mainly with the DuCV-1 genotype. These findings suggest that DuCV-1 may be more widespread globally than DuCV-2 is. However, these data vary in terms of reporting periods, and some countries have limited reported cases; therefore, it cannot be ruled out that DuCV-2 may also be prevalent in countries other than China, Vietnam, Taiwan, and Poland.

Compared to the scattered reports from various regions over a significant time span, a dense body of research originating from mainland China should better meet the fundamental requirements of epidemiological investigations. Chinese-origin studies significantly contribute to the broad epidemiological understanding of DuCV, portraying a more extensive spectrum of related data. For instance, during 2006–2007, the infection rate of DuCV in duck flocks in Shandong Province, China, was 33.29%, with samples drawn from 70 duck farms comprising 742 Cherry Valley ducks [34]. Moreover, an examination of 138 deceased duck samples from 18 distinct farms in southern China, conducted from March 2006 to December 2009, revealed a DuCV infection rate of approximately 35%, with the simultaneous identification of DuCV-1 and DuCV-2 strains [35]. This finding denoted the simultaneous prevalence of the two genotypes, a finding echoed by two concurrent studies. Specifically, six DuCV strains discerned from Cherry Valley ducks during 2007–2008 in China were categorized as both DuCV-1 and DuCV-2 [36]. Similarly, between 2008 and 2010, nine DuCV genomes were extracted from 90 deceased or afflicted ducks from diverse Chinese regions; these genes were also identified as DuCV-1 and DuCV-2 [37].

A decade later, from June 2018 to September 2019, 219 tissue samples were collected from deceased ducks across 20 farms located in central and eastern regions of China (Henan, Anhui, Zhejiang, and Fujian). Genetic assays revealed a high DuCV positivity rate of 87.21% in 191 samples across all farms [4]. Intriguingly, DuCV-1 was exclusively identified in all samples from these 20 duck flocks, with no instances of concurrent DuCV-1 or DuCV-2 infection recorded. Sequencing of 20 viral strains revealed sequence homology ranging from 82% (DuCV-2) to 99.7% (DuCV-1) when compared to DuCV sequences in GenBank. The principal variable regions are primarily situated within the open reading frames ORF2 and ORF3 and within the intergenic regions, with 15 strains ostensibly originating from recombination events between and within genotypes. During the same period, from 2018 to 2019, 848 samples from various duck species (Muscovy duck, Cherry Valley Pekin, Mallard) in southern and southwestern China (Guangdong, Guangxi, and Yunnan provinces) were examined, revealing a DuCV positivity rate of approximately 36.91% [22]. Nineteen complete viral genomes were sequenced, sharing a homology of 81.7–99.3% with 57 sequences in GenBank. The sequences from Guangdong and Guangxi Province were found to be mainly DuCV-1 and DuCV-2, respectively, with both of these genotypes coexisting in Yunnan Province.

Between April 2018 and March 2020, samples were collected from 69 diseased ducks on 32 duck farms across eight different regions in Anhui Province. The DuCV positivity rate was 36.2%, and DuCV-1d sequences were detected [38]. In a subsequent period, from October 2020 to August 2021, 270 diseased duck liver tissue samples were collected from four different areas in Guangdong Province. Sixty-nine samples tested positive (25.55%), with 16 strains belonging to the DuCV-1b genotype and four strains belonging to the DuCV-2c genotype. Two strains belonged to the DuCV-1a genotype, with DuCV-1b being more prevalent [39]. In 2022, DuCV-3 was discovered in Hunan Province.

In light of the above information and based on research reports in China, epidemiological surveys from various regions from 2006 to 2022 indicate that the prevalence of DuCV across various provinces in mainland China is gradually increasing each year. In particular, the DuCV positivity rate within East China ranged from 42.9%-58.46%, that in the Central-South region ranged from 2.38%-36%, that in the Southwest region approximately 43.09%, and that in the Northwest region ranged from 15.38%-38.46%. DuCV is widely prevalent across mainland China. Although epidemiological survey data have yet to be obtained for North and Northeast China, varying degrees of DuCV prevalence exist in other regions, with East China being the most severely affected region. Currently, the primary prevalent genotypes in mainland China are DuCV-1, DuCV-1a, 1b, and 2c, which exhibit varying prevalence levels. DuCV-1a is the main subtype prevalent in East China, while DuCV-1b is the main subtype prevalent in the Central-South region.

### The role of migratory birds in the transmission of DuCV

Notably, the incidence of DuCV infection is increasing annually, particularly in China’s coastal provinces, including Shandong, Jiangsu, Sichuan, Fujian, and Guangdong. This trend underscores that the DuCV incidence among duck populations manifests regional nuances. The prevalence pattern could be intricately tied to the migratory tendencies of wild ducks, as the eastern coastal areas of China are part of the East Asia-Australia migration route. Therefore, wild duck mating could be one of the key factors in the spread and prevalence of DuCV. Yuan et al. identified seven virus sequences in Guangdong Province, China, which formed a monophyletic group with circoviruses identified in wild ducks [39]. Additionally, Niu et al. collected 189 samples from 11 species of wild ducks and obtained four positive samples, for a positivity rate of 2.12%. These genes belong to both DuCV-1 and DuCV-2, indicating that the two genotypes of DuCV are widely distributed and common among migratory wild ducks [40].

Extending the scope beyond mainland China, Vietnam, Taiwan, and South Korea are also part of the East Asia-Australia migratory bird route. From April to October 2021, tissue samples were collected from duck farms and diseased birds across six provinces in northern Vietnam. Among the 130 duck samples, 56 (43.08%) with DuCV genomes were identified. Among the 38 farms investigated, 26 (68.42%) tested positive for DuCV. Phylogenetic examination of six sequenced DuCV genomes revealed that the prevailing strains in northern Vietnam closely agree with those in China, Taiwan, and South Korea, with four aligning with DuCV-1a, 1b, and 1c and the other two with DuCV-2b and 2c. These findings further substantiate the role of migratory birds in the dissemination of DuCV across Asia via migration routes. Moreover, the prevalence of DuCV is also affected by various factors, such as sample size, season, and even biosecurity policies.

## Clinical signs and pathological changes

### Single infection

DuCV infection often manifests subclinically, with no obvious clinical symptoms. After ducks are infected with DuCV, pathological changes in the immune system occur. The primary site of damage, the bursa of Fabricius, experiences considerable lymphocyte depletion characterized by necrosis and degeneration. Concurrently, pathological progression progresses to abnormal cell proliferation and atrophy within the spleen and thymus. The liver is most impacted by DuCV infection, as evidenced by yellowish-brown patch formations and a discernible reduction in bile production [3, 31]. A particular study investigated the protracted impact of DuCV, especially during the window from 8 to 32 days post-infection (dpi), on the development of duckling immune organs. The resulting immune disturbance is indicated by a reduction in the lymphocyte transformation rate (LTR), levels of IL-12, soluble CD4, soluble CD8, and IFN-γ and an increase in the level of IL-10 [2]. The association of DuCV with primary sclerosing cholangitis (PSC), a chronic cholestatic liver ailment, contradicts another layer of the pathological cascade. This disease exacerbates injury to bile duct epithelial cells, leading to progressive fibrous obstruction of bile ducts, a condition further exacerbated by lymphocyte infiltration [41]. By revealing the course of DuCV-1 infection, persistent and systemic infections occur. In the initial week following infection, DuCV DNA was detected across a spectrum of biological matrices—serum, rectal swabs, and various organs—underlying systemic invasion. Virion shedding continued unabated, with the virus remaining detectable even at 10 weeks post-infection. Among the organs, the spleen showed the highest average viral load, followed by the bursa of Fabricius, caecal tonsils, lungs, thymus, liver, and kidneys in descending order [3]. To date, there are no studies specifically targeting the pathogenicity of DuCV-2 and DuCV-3.

### Coinfection with other viruses

In routine duck breeding, the clinical manifestations and pathological alterations caused by DuCV often arise from coinfections with other pathogens. Among these, fowl adenovirus type 4 (FAdV-4) primarily targets chickens, inducing hydropericardium-hepatitis syndrome. However, an increase in FAdV-4-infected ducks, which commonly cooccur with DuCV and yield exacerbated clinical outcomes, has occurred in recent years. Animal models revealed that upon coinfection with DuCV and FAdV-4, the viral load of FAdV-4 markedly increased, amplifying its pathogenicity. Dissection revealed more severe pathological changes, such as liver rupture and hydropericardium, than did the mere liver swelling observed in singular infections. Importantly, the onset of hydropericardium occurs earlier in coinfected subjects than in singly infected individuals [42].

Beak atrophy and dwarfism syndrome (BADS), also known as short beak and dwarfism syndrome (SBDS), emerged in northern China in 2015 and is typically attributed to coinfection with DuCV and the novel goose parvovirus (NGPV). Approximately 85.7% of ducks were coinfected DuCV, and the affected populations exhibited growth retardation, beak atrophy, tongue protrusion, severe cases, and mortality [43]. Coinfection models reveal synergistic amplification of viral replication and pathogenicity between NGPV and DuCV. In such scenarios, viral loads of both NGPV and DuCV in ducks are significantly heightened during the latter stages of infection, predominantly involving the liver, kidneys, duodenum, spleen, and bursa of Fabricius. Concomitantly, the growth trajectory of ducks is markedly stunted, accompanied by immune organ atrophy, pallor, and liver necrosis, underlining the synergistic augmentation of pathogenicity in coinfected ducks [44].

In 2017, an outbreak of feather shedding syndrome (FSS) occurred in eastern China, where afflicted ducks exhibited novel clinical symptoms such as feather shedding and difficulty in feather plucking post-slaughter. Predominantly occurring in 4- to 5 week-old duck flocks, morbidity rates spanned 20% to 70%, with a 40% mortality rate among the infected birds. With a 70% coinfection rate of NGPV and DuCV, 4- to 5 week-old duck cohorts displayed pronounced FSS symptoms in response to this coinfection [21].

In addition to these documented instances, our clinical examinations revealed DuCV coinfections with other pathogens, such as duck enteritis virus, avian influenza virus (H9 subtype), duck hepatitis virus, egg drop syndrome virus, and duck tembusu virus (unpublished data). These coinfections lead to a spectrum of enhanced clinical manifestations. In summary, our findings underscore that DuCV orchestrates immunosuppression in ducks, rendering them more prone to other pathogenic invasions. Concurrently, DuCV potentiates the pathogenicity of coinvading pathogens, hastening disease evolution and considerably escalating both morbidity and mortality in affected ducks.

### Coinfection with bacteria

Coinfections of DuCV with bacteria are also commonly observed in clinical settings. Between 2011 and 2012, of 147 samples collected from 92 farms in South Korea, 32 (21.8%) tested positive for DuCV via PCR. Among these birds, 10.9% (16/147) had a single DuCV infection, 4.1% (6/147) were coinfected with *Riemerella anatipestifer*, and 5.4% (8/147) were coinfected with Salmonella Enteritidis. The likelihood of simultaneous infection with all three pathogens was 1.4% (2/147) [23]. Compared with DuCV-negative ducks, ducks positive for DuCV had higher rates of *R. anatipestifer* infection (23.48% vs. 8.28%) and *Escherichia coli* infection (16.19% vs. 4.85%) [34]. Another study revealed a greater incidence of *R. anatipestifer* and Salmonella Enteritidis in DuCV-positive duck farms (50%) than in DuCV-negative ones (26.1%) [23]. Furthermore, DuCV infection can significantly boost the pathogenicity and colonization ability of the avian pathogen *Escherichia coli* (APEC) within the host [2]. These findings suggest that DuCV infection increases susceptibility to bacterial infections. Therefore, controlling simultaneous bacterial coinfections is crucial when managing DuCV infections.

## Virus isolation and cultivation

Isolating and propagating DuCV or other avian viruses, such as duck plague virus (DPV), goose astrovirus (GAstV), and goose parvovirus (GPV), in established cell lines poses significant challenges. The primary method for DuCV isolation employs duck embryos; however, DuCV nucleic acids often become undetectable after merely four passages. Although effective isolation techniques for DuCV have yet to be identified, hindering in-depth exploration of DuCV, researchers continue to seek feasible methods. Three cell lines derived from Muscovy ducks—GE1.CR, AGE1.CR.pIX, and AGE1.CS—exhibit susceptibility to DuCV. Among these, the AGE1.CR.pIX cell line showed the highest yield and sensitivity during viral infections, with one infection cycle taking approximately 12 to 14 h [45]. Another study utilized peripheral blood mononuclear cells (PBMCs) from Pekin ducks for the propagation of DuCV [46]. While these cell lines can be infected by DuCV, they exhibit a relatively low viral yield. By optimizing viral infection protocols and/or modifying these cells, an enhanced viral yield may be achieved, potentially rendering these cell lines useful for vaccine development and production. However, further study of DuCV itself as well as its infection mechanisms with host cells is needed.

## Diagnostic and detection methods

Due to the subtle clinical symptoms of DuCV infection, diagnosis currently relies mainly on molecular diagnostic methods (Additional file 3). Standard methods such as polymerase chain reaction (PCR), real-time PCR, and nucleic acid probes are predominantly employed and are capable of efficaciously identifying the presence of DuCV or discerning various genotypes with a sensitivity reaching up to 10 copies/µL [47–50]. To further improve the sensitivity and specificity of DuCV detection in mixed infections, multiplex PCR and loop-mediated isothermal amplification (LAMP) have also been employed [51–53]. The recombinase-aided amplification-lateral flow dipstick (RAA-LFD) detection method allows reaction products to reach detectable levels in 20 min at 37  °C, facilitating rapid clinical diagnostics on farms [54]. Notably, DuCV shares high sequence homology with other circoviruses, and although these viruses do not infect ducks, sample contamination can occur. Therefore, it is essential to design primers and probes that avoid homologous regions to improve specificity. Research has shown that a quadruple tandem repeat sequence (QTR) exists between the rep and cap genes of the DuCV genome and can enhance mRNA stability [55]. The absence of the QTR notably diminishes virus transcription and replication. The uniqueness and evolutionary conservation of the QTR across genotype 1 and genotype 2 make it a molecular hallmark for DuCV genotyping.

Serological detection techniques for DuCV predominantly involve ELISA, typically using the DuCV Cap protein as the coating antigen. Given that the N-terminal NLS region of the Cap protein hinders protein expression, this region may be omitted or codons optimized [56, 57]. Recent studies have developed a DuCV capsid protein-specific monoclonal antibody (mAb) that can recognize the exposed epitope ^144^IDKDGQIV^151^ on the viral surface, potentially having diagnostic and research applications [58]. In addition to Cap, Rep or ORF3-encoded proteins, even specific peptides can also serve as antigens. However, caution is needed when considering the low cross-reactivity caused by antigenic variation between different genotypes and nonspecific reactions possibly generated with other viral antisera.

## Vaccines and antiviral drugs

The absence of an in vitro cell culture system for large-scale propagation of DuCV has impeded the availability of commercial vaccines, highlighting the need for advancing the research and development of novel vaccines and antiviral compounds. Some research has utilized PBMCs for the isolation and propagation of DuCV, and an inactivated vaccine has been developed. Ducks vaccinated with this inactivated vaccine did not exhibit feather abnormalities, growth inhibition, or dwarfism, and no lesions or lymphocyte apoptosis were observed in the bursa of Fabricius, spleen, or thymus. The resulting antisera showed high neutralizing antibody titres [46]. However, the labour-intensive nature of isolating PBMCs poses a significant bottleneck for the large-scale production of DuCV inactivated vaccines.

Infectious clones could serve as candidate vaccines for DuCV. Research has led to the creation of an infectious clone of DuCV and the study of the infection characteristics of the rescue virus in ducks. The data revealed that while the rescue virus closely mirrored the parent virus, it exhibited diminished pathogenicity and replicative ability [59]. In addition, infectious clones could unveil a spectrum of insights into the pathogen’s transmission routes, life cycle, and replication mechanisms, thereby illuminating the disease’s origins and transmission modes. They also enable the exploration of pathogen–host interactions, disease mechanisms, and immune evasion strategies. By constructing infectious clones with specific gene deletions or mutations, the role of these genes in the pathogenic process can be evaluated, contributing to the understanding of disease progression, host immune responses, and the identification of intervention strategies and targets.

Subunit vaccines, which are generally composed of specific virus components or antigens, are another potential vaccine candidate for DuCV. These approaches are characterized by high safety, good stability, low risk, and strong customizability. The Cap protein of DuCV has high immunogenicity and contains the main antigenic epitopes of DuCV, making it the preferred antigen for subunit vaccines. In addition, the Cap protein can self-assemble into virus-like particles (VLPs), mimicking the morphology of wild-type viral particles to better stimulate the immune response of the host, with great potential as a candidate vaccine [60].

While effective commercial drugs for combating DuCV infection are currently unavailable, certain medications may mitigate DuCV pathogenicity, thus offering some control over infection and curbing economic losses. Certain natural drug components can target the immune system of the host, playing a role in immune regulation and, to some extent, inhibiting infection by the virus. Several studies have identified compounds such as Astragalus polysaccharide (APS) and pine pollen polysaccharide (PPPS), which are derived from Astragalus and pine pollen, respectively; these compounds exhibit efficacy in enhancing duck immunity, reducing viral load, and alleviating damage to immune organs caused by DuCV. Additionally, APS and PPPS significantly reduced DuCV-induced apoptosis of peripheral blood lymphocytes [61]. Antibody therapy, another potential treatment for viral infections, has also shown promise. The administration of recombinant duck IFN-α markedly alleviated the clinical symptoms of immune organ atrophy and immune suppression triggered by DuCV infection. Moreover, combined treatment with recombinant duck IFN-α and specific polyclonal antibodies successfully halted DuCV infection 13 days post-treatment under experimental conditions, demonstrating superior efficacy in inhibiting DuCV infection compared to single treatments [62].

## Prevention and control

Due to the absence of vaccines and effective therapeutic drugs, current prevention and control strategies for DuCV infection predominantly revolve around bolstering farm management and enforcing stringent flock quarantine measures. Unlike other avian diseases, DuCV neither directly causes duck mortality nor causes typical clinical symptoms. Instead, it impairs the immune response in ducks, facilitating subsequent infections and escalating the pathogenicity of other microbes. Consequently, it is imperative for duck farmers to focus on enhancing flock immunity and averting mixed infections to prevent substantial economic losses resulting from mass mortality. Studies indicate that by 8 dpi, DuCV stably persists in various organs, underscoring the necessity for early prevention and control measures against this virus in farming operations [2].

Recommended management practices include routine disinfection of poultry houses, for instance, through fumigation with a mixture of potassium permanganate and 30% formalin. For waste disposal, a 10% formalin solution is recommended. Ducks succumbing to abnormal causes should be disposed of, while the remaining flock in the same shed should be isolated and thoroughly disinfected. Basic vaccinations, including inoculations against avian influenza, duck plague, duck hepatitis, and FAdV, are crucial for flocks. Ensuring a steady supply of fresh and nutritious feed is fundamental to bolstering flock resistance. Regular health monitoring of the flock and routine testing are indispensable; ducks exhibiting abnormal symptoms should be promptly isolated and examined. Implementing thorough screening of duck flock diseases can help to prevent the spread of and minimize losses due to epidemics.

## Conclusions and future directions

Over the two decades since its discovery, the prevalence of DuCV has steadily increased globally, intensifying over time. A more alarming issue is the insufficient attention given to DuCV infections, which is mirrored by the sparse related publications in the PubMed database. Although solitary infections caused by DuCV do not directly lead to disease symptoms, its propensity to impair the immune system and facilitate infection by other pathogens poses a serious threat to the duck farming industry. Hence, escalating research efforts and fortifying prevention and control measures against DuCV are paramount.

The majority of the research on DuCV is currently from China, revealing an escalating epidemic situation. Several factors may drive this trend. First, as of 2020, data from the Food and Agriculture Organization (FAO) indicated that approximately 89% of the world’s duck population (estimated at 1.15 billion) is located in Asia. Along with Vietnam, Bangladesh, and Indonesia, China hosts the largest duck populations. China inherently faces a greater likelihood of outbreak because of its stature as the world’s largest duck-farming nation. Second, the diverse duck farming models in China, particularly small-scale family farms, often involve rudimentary management practices, which exacerbate the challenge of virus spread control. Third, the lack of effective prevention and control measures for DuCV, compounded by potential viral mutations, could amplify infectivity. Fourth, the scarcity of related research and reports from other countries indirectly highlights the severity of the epidemic in China.

The current discourse on DuCV research reveals several significant gaps. First, the global scale of inquiries remains minimal, despite sporadic surveys being conducted across different countries and regions over the years. Given the often latent nature of DuCV infections, an absence of reported cases in certain locales does not necessarily denote a lack of DuCV infection but rather may suggest inadequate testing. Second, although extensive research has evaluated the molecular epidemiology and genetic variation of DuCV, there is a pressing need for further exploration of various facets of DuCV, such as viral invasion, replication, pathogenic mechanisms, host interactions, and immune responses. Third, while several diagnostic methods have been developed, their feasibility and accuracy for field applications necessitate validation. Finally, continuous in vitro passages of DuCV have not been performed, and effective vaccines or drugs targeting DuCV have yet to be formulated.

Addressing these issues demands a multipronged approach. First, obtaining additional data on the global prevalence of DuCV is vital for obtaining a nuanced understanding of its transmission dynamics, which in turn can inform effective prevention strategies. Second, more thorough investigations into the pathogenic mechanisms and host immune responses of DuCV are warranted, laying a theoretical foundation for vaccine and drug development. Given the commonalities within the *Circoviridae* family, insights may be gleaned from established research paradigms and findings on PCV. Furthermore, continuous optimization of existing diagnostic methods, coupled with the development of rapid, simple, and precise diagnostic techniques suitable for field use, is essential. Finally, accelerating the development and clinical validation of DuCV vaccines and drugs is imperative. Concurrent efforts to fortify disease prevention and control within the duck farming industry will significantly mitigate the risk of DuCV transmission. As we look forward, advancing scientific technology will likely yield a deeper understanding of DuCV, alongside the development of effective preventive measures, thereby significantly enhancing the control of DuCV infections and securing the health and growth trajectory of the duck farming industry.

### Supplementary Information

**Additional file 1: Epidemiological investigations of DuCV infection worldwide (2003–2023).**

**Additional file 2: A comparison of homologous features in genomes and proteins among various circoviruses.**

**Additional file 3: Different molecular diagnostic methods for DuCV detection.**

## Data Availability

The data supporting the conclusions of this article are included within the article. Additional datasets used and/or analysed during the current study are available from the corresponding author upon reasonable request. Any materials utilized in this review are publicly accessible in the referenced publications.
