# Supplementary material for: From obscurity to urgency: a comprehensive analysis of the rising threat of duck circovirus
Source: Vet Res. 2024 Jan 26;55:12. doi: 10.1186/s13567-024-01265-2 (PMC10811865; doi:10.1186/s13567-024-01265-2)
Supplement: Supplementary file 1 — Additional file 1: Epidemiological investigations of DuCV infection worldwide (2003–2023). [file 13567_2024_1265_MOESM1_ESM.docx]

**Table S1. Epidemiological investigations of DuCV infection worldwide (2003-2023)**

| **Country** | **Region** | **Species** | **Genotype** | **Reported Year** | **Infection Rate** | **Genbank ID** | **Refer.** |
| --- | --- | --- | --- | --- | --- | --- | --- |
| **Germany** | Brandenburg | Mulard | DuCV-1a | 2003 | N/A | AY228555 | [6] |
| **Hungary** |  | Mulard, Peking and Muscovy ducks | N/A | 2005 | 84.16%  (85/101 samples)  94.59%  (35/37 flocks) | AJ964962  （rep gene） | [25] |
| **China** | Taiwan | Muscovy, mule, and Pekin ducks | DuCV-2b | 2006 | 38.24%  (13/34 samples) | AY394721  DQ166836--166838 | [26] |
|  | Fujian province | Muscovy duck | DuCV-2c | 2008 | 79.07%  (34/43 samples)  83.33%  (10/12 farms) | EF370476 | [63] |
|  | Fujian province | Domestic duck | DuCV-1a | 2008 | N/A | EF451157 | [64] |
|  | Shandong province | Cherry Valley ducks | N/A | 2009 | 33.29%  (247/742 samples) | N/A | [34] |
|  | Shandong province | Cherry Valley ducks | N/A | 2010 | 15.24%  (246/1614 samples) | N/A | [56] |
|  | Fujian province | Muscovy,  mallard, Pekin ducks | DuCV-1a  DuCV-2a | 2011 | 12.85%  (23/179 samples) | EU344802--EU344807  EU499309--EU499311 | [7] |
|  | Fujian, Zhejiang and Guangdong provinces | Muscovy, Mule, Cheery, Mulard and Pekin ducks | DuCV-1a  DuCV-2c | 2011 | 35.5%  (49/138 samples) | GQ423740-GQ423747  GU168779 | [35] |
|  | Hebei, Jiangsu, Shandong provinces and Beijing | Pekin duck | DuCV-1b | 2011 | 10%  (10/90 samples) | HM162345--HM162353 | [37] |
|  | Shandong province | Cherry Valley duck | DuCV-1a, 1b  DuCV-2c | 2012 | N/A | EU022374  EU022375  GU131340--GU131343 | [36] |
|  | Sichuan province | N/A | DuCV-2c | 2013 | 2%  (1/50 samples) | JX499186 | [8] |
|  | Yunnan province | Muscovy duckling | DuCV-1d | 2016 | N/A | KR491946 | [9] |
|  | Shandong, Jiangsu and Anhui provinces | Cherry Valley duck and Mule ducks | DuCV-1a, 1b | 2018 | 82.35%  (140/170 samples) | MF627687-- MF627690 | [65] |
|  | Northeast China | Mallard, Green-winged Teal, and Falcated ducks | DuCV-1a, 1b  DuCV-2b, 2c | 2018 | 2.12%  (4/189 samples) | KU844855--KU844858 | [40] |
|  | Zhejiang, Henan, Fujian, and Anhui provinces |  | DuCV-1a, 1b,  1d | 2020 | 87.2%  (191/219 samples) | MN928792--MN928811 | [4] |
|  | Guangdong, Yunnan and Guangxi provinces | Mulard, Cherry Valley, Muscovy, and Mallard ducks | DuCV-1a, 1b, 1d. DuCV-2c | 2020 | 36.91%  (313/848) | MK814571--MK814577  MK814584--MK814589  MK814578--MK814583 | [22] |
|  | Anhui province | N/A | DuCV-1d | 2020 | 16.13%  (5/31 samples) | MT646346 | [48] |
|  | Shandong, Jiangsu, and Anhui provinces | Cherry Valley duck | DuCV-1  DuCV-2 | 2020 | 78.89%  (426/540 samples) | N/A | [21] |
|  | Anhui province | N/A | DuCV-1b, 1d  DuCV-2c | 2021 | 36.2%  (25/69 samples) | MT646346-MT646350 | [38] |
|  | Guangdong provinces | Domestic duck | DuCV-1a, 1b  DuCV-2c | 2022 | 25.6%  (69/270 samples) | ON227545  ON227537  ON227539  ON227536  ON227555-ON227557 | [39] |
|  | Shandong and Jiangsu provinces | Cherry Valley ducks | DuCV-1b | 2023 | 33.33%  (4/12 samples) | OQ657183--OQ657186 | [66] |
| **United States of America** | New York | Pekin duck | DuCV-1b | 2007 | 6.45%  (2/31 flocks) | NC_007220, DQ100076 | [27] |
| **South Korea** | N/A | Pekin duck | DuCV-1b | 2013 | 21.9%  (32/147 samples) | JQ740360--740363  KC851804--851823 | [29] |
|  | Gyeonggi, Chungnam, Chungbuk, Chonbuk and Chonnam | Pekin duck | N/A | 2014 | 21.8%  (32/147 samples) | N/A | [23] |
| **Poland** | Wrocław | Velvet scoter  (Melanitta fusca) | DuCV-2a | 2015 | 17.39%  (4/23 samples) | KP943594 | [33] |
| **Brazil** | State of Santa Catarina | Pekin duck | DuCV-1b | 2020 | 35% | MT318126 | [30] |
| **Vietnam** | Hanoi, Haiduong, Thainguyen, Bacgiang, Thaibinh and Hungyen province | N/A | DuCV-1b  DuCV-2b, 2c | 2022 | 43.08%  (56/130 samples)  68.42%  (26/38 farms) | OM176552--OM176557 | [20] |
| **Great Britain** | N/A | Aylesbury duck | DuCV-1 | 2022 | 54.54%  (18/33 samples) | N/A | [31] |

Note: Nearly all samples come from sick or dead animals, which has resulted in a very high reported positivity rate.

**Additional references**

63. Jiang S-j, Zhang X-x, Liu S-n, Wang Y, Kong Y-b, Wei X-l, Sun Y-n, Zhao Q (2008) PCR detection and sequence analysis of duck circovirus in sick Muscovy ducks. Virologica Sinica 23:265-271

64. Fu GH, Cheng LF, Shi SH, Peng CX, Chen HM, Huang Y (2008) [Genome cloning and sequence analysis of duck circovirus]. Bing Du Xue Bao 24:138-143

65. Li P, Li J, Zhang R, Chen J, Wang W, Lan J, Xie Z, Jiang S (2018) Duck "beak atrophy and dwarfism syndrome" disease complex: Interplay of novel goose parvovirus-related virus and duck circovirus? Transbound Emerg Dis 65:345-351

66. Zhang T, Liu N, Zhang L, Jiang W, Fan X, Wang X, Miao R, Zhai X, Wei L, Jiang S, Jiao P (2023) Research Note: Complete genome cloning and genetic evolution analysis of four Cherry Valley duck circovirus strains in China in 2022. Poult Sci 102:102920
