# Supplementary material for: From obscurity to urgency: a comprehensive analysis of the rising threat of duck circovirus
Source: Vet Res. 2024 Jan 26;55:12. doi: 10.1186/s13567-024-01265-2 (PMC10811865; doi:10.1186/s13567-024-01265-2)
Supplement: Supplementary file 2 — Additional file 2: A comparison of homologous features in genomes and proteins among various circoviruses. [file 13567_2024_1265_MOESM2_ESM.docx]

**Additional file 2 A comparison of homologous features in genomes and proteins among various circoviruses**

| Different circoviruses | GenBank ID | Genome | | Rep protein | | | Cap protein | | |
| --- | --- | --- | --- | --- | --- | --- | --- | --- | --- |
|  |  | Length (nt) | Homology | | Length (aa) | Homology | | Length (aa) | Homology |
| DuCV-1a | MF627690 | 1995 | N/A | | 292 | N/A | | 257 | N/A |
| DuCV-1b | HM162351 | 1996 | 96.00% | | 292 | 98.60% | | 257 | 99.20% |
| DuCV-1c | GU014543 | 1995 | 94.80% | | 292 | 97.90% | | 257 | 96.10% |
| DuCV-1d | KR491946 | 1987 | 94.50% | | 292 | 99.00% | | 257 | 94.90% |
| DuCV-2a | EU344805 | 1988 | 84.80% | | 292 | 96.20% | | 257 | 87.50% |
| DuCV-2b | KP229377 | 1988 | 83.20% | | 292 | 93.80% | | 257 | 87.90% |
| DuCV-2c | ON227555 | 1988 | 82.80% | | 292 | 93.80% | | 257 | 86.00% |
| DuCV-3 | OP432310 | 1755 | 70.80% | | 293 | 88.40% | | 245 | 49.20% |
| Goose circovirus (GoCV) | AF536941 | 1821 | 67.00% | | 293 | 83.20% | | 250 | 47.10% |
| Swan (Cygnus olor) circovirus | EU056309 | 1783 | 66.30% | | 293 | 78.40% | | 251 | 55.50% |
| Wigeon (WigFec) circovirus 1 | MZ604582 | 1723 | 55.60% | | 288 | 63.90% | | 254 | 27.30% |
| Porcine circovirus 3 (PCV-3) | KX458235 | 2000 | 44.20% | | 165 | 50.00% | | 214 | 25.50% |
| Porcine circovirus 1 (PCV-1) | AF071879 | 1758 | 44.10% | | 312 | 44.10% | | 230 | 25.70% |
| Finch circovirus | DQ845075 | 1962 | 43.20% | | 291 | 50.50% | | 249 | 21.10% |
| Bovine circovirus (BoCV) | AF109397 | 1768 | 42.90% | | 314 | 44.50% | | 159 | 23.10% |
| Porcine circovirus 4 (PCV-4) | MW238796 | 1770 | 42.80% | | 296 | 48.60% | | 228 | 26.10% |
| Psittacine beak and feather disease virus (BFDV) | AF080560 | 1993 | 42.70% | | 289 | 49.00% | | 247 | 22.00% |
| Porcine circovirus 2 (PCV-2) | KX814348 | 1767 | 42.50% | | 314 | 44.10% | | 234 | 23.40% |
| Columbid circovirus (CoCV) | AF252610 | 2037 | 42.40% | | 317 | 50.90% | | 273 | 24.30% |
| Raven circovirus | DQ146997 | 1898 | 42.30% | | 291 | 49.10% | | 243 | 25.00% |
| Starling circovirus | DQ172906 | 2063 | 42.30% | | 289 | 49.70% | | 276 | 25.80% |
| Pigeon circovirus (PiCV) | OR611917 | 2037 | 42.10% | | 317 | 51.20% | | 272 | 24.10% |
| Bat (Siksparnis) circovirus | KX756996 | 1761 | 41.60% | | 312 | 43.40% | | 235 | 21.40% |
| Canary circovirus (CaCV) | AJ301633 | 1952 | 41.40% | | 290 | 49.10% | | 250 | 23.50% |
| Barbel circovirus | GU799606 | 1957 | 41.30% | | 319 | 43.80% | | 166 | 25.30% |
| Wigeon (WigFec) circovirus 2 | MZ604590 | 1821 | 41.00% | | 304 | 46.70% | | 215 | 27.10% |
| Gull circovirus | DQ845074 | 2035 | 40.90% | | 305 | 47.00% | | 245 | 22.40% |
| Catfish (Silurus glanis) circovirus | JQ011377 | 1966 | 40.70% | | 314 | 44.00% | | 227 | 19.20% |
| Canine circovirus | JQ821392 | 2063 | 29.47% | | 303 | 47.90% | | 270 | 19.90% |
| Bear (Ursus americanus) circovirus | MN371255 | 2054 | 20.60% | | 286 | 42.60% | | 259 | 19.30% |
