# Supplementary material for: From obscurity to urgency: a comprehensive analysis of the rising threat of duck circovirus
Source: Vet Res. 2024 Jan 26;55:12. doi: 10.1186/s13567-024-01265-2 (PMC10811865; doi:10.1186/s13567-024-01265-2)
Supplement: Supplementary file 3 — Additional file 3: Different molecular diagnostic methods for DuCV detection. [file 13567_2024_1265_MOESM3_ESM.docx]

**Table S3. Different molecular diagnostic methods for DuCV detection**

| **Method** | **Sequence of Primers** | **Target Sequence** | **Product Length** | **Sensitivity** | **Reported Year** | **Reference** |
| --- | --- | --- | --- | --- | --- | --- |
| Real-Time PCR  (SYBR Green I) | Forward: CCCGCCGAAAACAAGTATTA  Reverse: TCGCTCTTGTACCAATCACG | *rep* gene of AY228555  541-770 nt | 230 bp | 13×10^3^ copies/μL | 2005 | [25] |
| Real-time PCR (SYBR Green I) | DuCVp1: TGTTATCTTTGGGCGTGG  DuCVp2: CATTTCCCGAGTAACCGTC | Genbank: EF451157  459-649 nt | 191 bp | 1.31×10^2^ copies/μL | 2011 | [47] |
| Loop-mediated isothermal amplification (LAMP) | Outer primers:  F3: AGTTBTGCACGCTCGACAAT  B3: GTCGACTCTTTGGMGCAATA  Inner primers:  FIP: TTCAGGAATCCCTGAAGGTGATCGTCGGMGAGGAVAAGG  BIP: GCGMGAGCTGCCGCCCTTCTTCVTCAGATCCCCGG  Loop primers:  LoopF: AGGYGTVCCVTTCGCGC  LoopB: AGGAAGAGCCTGGCTCTC  (M = A or C; V = A or C or G; B = T or G or C.) | Genbank: DQ100076  135–339 nt  236–309 nt  186–285 nt | 205 bp  74 bp  100 bp | 20 copies/μL | 2014 | [53] |
| GeXP-based multiplex PCR | AGGTGACACTATAGAATATGCKCCAAAGAGTCGACATA  GTACGACTCACTATAGGGACAAAYGCATAACGGCTCTTTCC  Universal tag sequences are underlined  (K=G or T, Y=C or T) | *rep* gene of JX241046 | 300 bp | 10^2^-10^3^ copies/μL | 2015 | [51] |
| Real-time PCR (SYBR Green I) | F: GTATCACTGACCGTTACC  R: GCTCTTTGTTGCTAGTTATG | *rep* gene of DQ10076 | 93 bp | 1×10^1^ copies/μL | 2020 | [48] |
| Recombinase-aided amplification-lateral flow dipstick (RAA-LFD) | DuCV-F: AAAGAGCCGTTATGCATTTGAATTTCCCGCCG  DuCV-R: Biotin-ACGGTCGGTAATTCTCAGCAAATCATCATACG  DuCV-T: FAM-ATTACAAACCACGCGGGAAGTGGTGGGACGG-  TTA/idSp/TCGGGAAATGACGTAG | *rep* gene  564-595 nt  686-717 nt  605-655 nt | 154 bp | 10^2^ copies/μL | 2021 | [54] |
| Real-time PCR (TaqMan) | DuCV-qPCR-F: CCGAAAACAAGTATTACAAAC  DuCV-qPCR-R: GTACCCAACCATAAAAGTC  Probe: DuCV-qPCR-P: FAM-TTCCCGAGTAACCGTCCCAC-BHQ1 | *rep* gene of  MN822911 | 93 bp | 39.4 copies/μL | 2021 | [49] |
| Real-time PCR (TaqMan) | F: TATGTTATCTTTGGGCGTGG  R: ACGACTACGTCATTTCCCGA  Probe: FAM-CACGCGGGAAGTGGTGGGACG-BHQ1. | *rep* gene of MN068360  457-659 nt | 203 bp | 2×10^1^  copies/μL | 2021 | [50] |
| Real-time fluorescence-based recombinaseaided amplification (RF-RAA) | DuCV-1-F: CCTCTGATCTGGCCGAAGCGACATCCGCTGT  DuCV-2-F: CTGTGATGGCTGGCGTCCCGCTGACTGAGGTG  DuCV-3-F: CCGCTGACTGAGGTGGCCCGGAAGTTCCCC  DuCV-1-R: TGCCGGGAGGACCAATCAGAACGATGACTT  DuCV-2-R: AATTCAAATGCATAACGGCTCTTTCCGGTG  DuCV-3-R: CCGCGTGGTTTGTAATACTTGTTTTCGGCG  Probe：DuCV-T: ATGTTCTTTGGGCGTGGCCTGGAACGCC  /i6FAMdT//idSp/CG/iBHQ1dT/CACCTGATCGTTGAG | *rep* gene  377-407  404-435  421-450  530-557  558-587  591-620  458−507 | 137-244 bp | 10^1^ copies/mL | 2021 | [67] |

**Additional references**

67. Li X, Wang C, Zhang Z, Wang C, Wang W, Zhao Z, Li J, Shang Z, Lv J, Zhang T (2022) Fast detection of duck circovirus by real-time fluorescence-based recombinase-aided amplification. Poult Sci 101:101707
